# Knowledge and attitude of women towards the legalization of abortion in the selected town of Ethiopia: a cross sectional study

**DOI:** 10.1186/s12978-018-0634-0

**Published:** 2018-11-21

**Authors:** Tilahun Fufa Debela, Misgun Shewangizaw Mekuria

**Affiliations:** 1 Institute of Health, Faculty of public health, Department of Health Economics Management and Policy, Jimma University, Jimma, Ethiopia; 2 College of Medicine and Health Sciences, Department of Public Health, Arba Minch University, Arba Minch, Ethiopia

**Keywords:** Abortion, Legalization, Women in reproductive age, Ethiopia

## Abstract

**Background:**

Unsafe abortion contributes to maternal deaths 13% globally and 25–35% of Ethiopia. By considering the problem of unsafe abortion, Ethiopia amended a law that permits abortion under certain circumstances. However, the country liberalized the service, women are still not using it. Therefore, the possible reason might be a lack of knowledge and attitude is a barrier that hinders women to use safe abortion.

**Methods:**

A community-based cross-sectional study was conducted in Arba Minch town from January 02 to 17, 2017. Women in the reproductive age groups (15–49) who reside in the town for more than six months were included in the study. The sample size was determined using a single population proportion formula. Five kebeles were selected using the lottery method from 11 kebeles. The proportional allocation of the sample was done for each kebeles. Data were collected using a structured questionnaire. Binary and multiple logistic analyses were carried out to identify factors associated with knowledge & attitude toward legalization of abortion.

**Result:**

A total of 576 women were responded to the question. The finding of our study showed that only 23.4% of women have knowledge about the legalization of abortion. Of all the respondents 323(56%) prefer abortion on demand to be legalized while about 241 (41.9%) do not prefer to be legalized. Again about 57% of women believe that women can use it but the rest 43% believe even if allowed women do not use it. From all participants, 59% don’t want to use by themselves and also, 53.3% don’t think that women would have the right to use the service or terminate their pregnancy even if the pregnancy fulfill the criteria. Ethnicity, marital status, and family size were the factors significantly associated with knowledge. Again, educational status, marital status and having knowledge about the legalization of abortion were a statistically significant association with the attitude.

**Conclusion:**

The study indicated that knowledge of women toward the legalization of abortion was low but more than half of respondents prefer abortion on demand to be legalized.

## Plain English summary

Unsafe abortion contributes about 13% of the global burden of maternal mortality and up to 25–35% of maternal deaths in Ethiopia. Sixty nine percent of Ethiopian women who experienced termination of pregnancy used unsafe abortion practices.

The aim of this study was to assess the knowledge and attitude of women towards legalization of abortion and its associated factors. The data were collected voluntarily and women who were critically ill, unable to talk or listen were excluded from the study. To measure knowledge; first, we asked whether women were aware the current abortion law of Ethiopia; if they answered yes, we continued to ask the legal prerequisites in Ethiopia to interrupt pregnancy. Knowledge of women toward the legalization of abortion was measured by seven closed-ended questions. The answers for the seven questions were aggregated out of seven. Those respondents who score above the median knowledge level (median knowledge score = 4) were considered as having good knowledge and those who score less than the median score were classified as having poor knowledge toward abortion legislation.

The attitude of women toward abortion legislation was measured by asking five closed-ended questions with both positive and negative responses. Those women who agreed or answer positively, considered as positive attitude and those respondents disagreed or negatively responded were considered as a negative attitude.

Of the 576 respondents: only 23.4% of women have good knowledge and 56% prefer abortion on demand to be legalized. Forty-three percent of women do not want to use the service even if it was legalized. And, 53.3% of women don’t think that women would have the right to use the service even if the pregnancy fulfills the legal criteria. Knowledge of abortion legislation differs among ethnic group, marital status, and households with different family size. Again, level of education, marital status, and knowledge of women about legislation of abortion were the associated factors for the attitude of women.

In conclusion, knowledge of women toward the legalization of abortion was low but more than half of respondents prefer abortion on demand to be legalized.

## Background

Maternal mortality is a public health problem in the world, especially in developing countries. Each year more than half a million maternal death happen in the world. From this, 99% occur in developing countries [1]. Sub Saharan Africa and South Asian alone accounts for 84% global maternal deaths [2, 3]. There are many factors contributing to maternal deaths, from these hemorrhages, infection during and after delivery and also unsafe abortion are among the leading cause of maternal death [4]. Unsafe abortion alone contributes about 13% of the global burden of maternal mortality [5]. According to World Health Organization, every year greater than 42 million pregnancies are terminated due to various reasons; from that, approximately 20 million are due to unsafe abortions and it is estimated about 80, 000 worldwide deaths from it [6, 7].

In Ethiopia, the number of maternal deaths associated with complication of pregnancy and delivery is among the highest in the world [5]. In Ethiopia, the ratio of maternal mortality (MMR) is 412 per 100,000 live births [8]. Several studies indicate that unsafe abortion accounts for up to 25–35% of maternal deaths in Ethiopia [9, 10]. Unsafe abortion complication found to be significant public health problems in Ethiopia, accounting for the higher proportion of maternal morbidity, mortality and gynaecological admissions [7]. It can be prevented and reduced by expanding and improving family planning services and choices. With a low modern contraceptive prevalence rate (4.8%) and a high total fertility rate (6.8–7%), a large number of Ethiopian women faced unwanted pregnancies [5]. Sixty nine percent of Ethiopian women who experienced termination of pregnancy used unsafe abortion practices rather than medically supervised abortion [2, 5]. The reason behind might be the lack of knowledge and attitude of women toward the legalization of abortion.

Ethiopia amended abortion law in May 2005 under certain conditions. Abortion is now legal in cases of rape, incest or fetal impairment. In addition, a woman can legally terminate a pregnancy if her life or physical health is in danger, if she has physical or mental disabilities, or if she is a minor who is physically or mentally unprepared for childbirth [9, 11, 12].

Knowledge about abortion law among women is very important because it has implications for access to legal abortion services [13]. As outlined in the WHO guideline on safe abortion, the proportion of women with correct knowledge of the legal status of abortion are both indicators for measuring access to information about safe abortion [14]. Even when safe, legal abortion services are available, women who lack accurate information about the law may seek unsafe abortion because they do not know that they are eligible for the service or do not know the legal requirements for obtaining an abortion [15]. Knowledge alone is not guarantee to use any service; but the attitude determines.

Research on knowledge of abortion law and attitude of women towards the law may help to inform policy makers and education planners in Ethiopia. Unfortunately, not much research has been conducted in this area among the women in the country. The aim of this study was to investigate knowledge and attitude of women toward the abortion law. Furthermore this study also identifies the associated factors influencing knowledge and attitude of women toward the legalization abortion.

## Methods

### Study design and setting

A community based cross-sectional study design was conduct from January 02 to 17, 2017 in Arba Minch town. The Town is found 465 km from Addis Ababa (the capital city of Ethiopia) to the south. The town has 11 kebeles (the lower administrative unit of Ethiopia). According to population projection of the 2007 national census conducted by the central statistics agency of Ethiopia (CSA), there was an estimated population of 110,104 of whom 53,951 were men and 56,153 were women. From this, 21,360 were reproductive age women. There were 19,000 households in the town during the data collection period.

### Study participants

The study population was women in reproductive-age who were living in the town for more than six months in the randomly selected kebeles of the town. The data were collected voluntarily and women who were critically ill, unable to talk or listen were excluded from the study.

### Sample size and sampling method

The required sample size was determined using a single population proportion formula. The assumptions considered were; proportion (p) of 50%, a margin error of 5%, a design effect of 1.5 and none response rate of 10%. Accordingly, the sample size was: n = (1.96)^2^ × 0.5 (1–0.5)/ (0.05)^2^; *n* = 384, and by considering 10% no response rate and a design effect of 1.5 the total sample size was 633. A multistage sampling technique was used. Five kebeles out of 11 kebeles were randomly selected using the lottery method. List of reproductive age women was extracted from a community-based intervention for action (CBIA) data in the selected kebeles which were collected by health extension workers. The calculated sample size was proportionally allocated to each kebele. To have individual study subjects, systematic sampling method was employed during data collection with K value of 4 (*N* = 2591 and *n* = 633 i.e. every 4th from the registration). The first woman was selected by lottery method.

### Data collection procedure

Data were collected using field-tested structured questionnaire. The questionnaire was developed after reviewing related literatures. The questionnaire has different sessions such as socio-demographic characteristics of respondents, 7 abortion history items, 5 attitude items and 7 items on knowledge questions. The questionnaire prepared in English was translated to Amharic (local language) and back to English in order to maintain consistency. Five data collectors those speak the local language (Amharic) collected the data with two supervisors.

### Data analysis

Data were entered into EpiData v3.1, exported to SPSS version 21 and cleaned to check for completeness and missing values. Descriptive statistics such as frequencies and summary statistics were used to describe the study population in relation to relevant variables. In binary logistic regression, both bivariate and multivariate analyses were carried out. All variables were entered into the bivariate analysis to identify the association between dependent and independent variables. Those explanatory variables with a *p*-value < 0.25 in the crude analysis had been used for multivariate analysis. In multivariate analysis, those variables with the p-value < 0.05 were considered as predictors of the legalization of abortion care.

### Measurements

Knowledge of abortion legalization was measured by asking seven abortion legislation questions. Questions were developed based on reviewing the Ethiopian legislation for abortion and other similar studies [10, 16–18]. First, women asked whether they aware about the current abortion law of Ethiopia; if the woman answered yes, we continued to ask the legal prerequisites in Ethiopia to interrupt pregnancy to know their knowledge level. To assess knowledge of the abortion law, seven closed-ended questions were used. The answers for these seven questions were aggregated out of seven. Those respondents who score above the median knowledge level (median knowledge score = 4) were considered as having good knowledge and those who score less than the mean score were classified as having poor knowledge of abortion legislation.

The attitude of women toward abortion legislation was measured by asking five closed-ended questions with both positive and negative responses. Those women who agreed or answer positively, considered as a positive attitude and those respondents disagreed or negatively responded were considered as a negative attitude.

### Data quality management

The questionnaires were pretested outside the study area. After the pretest, the questionnaire was reviewed for appropriateness of wording; clarity of both contents and whether instructions elicited is going with responses. Data collectors were trained for one day to be familiar with the data collection tool. Editing and sorting of the questionnaires were done to determine the completeness and consistency of data every day during the data collection. The completed questionnaires were cross-checked and made a correction on daily basis.

## Results

### Socio-demographic characteristics

A total of 576 women were interviewed from five kebeles. The overall response rate was 91%. One hundred sixty seven (29%) of the respondents were in the age group of 35–39 with the mean age of 34.48 + 5.43. Forty-five percent of women were Gamo in ethnicity while 27.9% were Konso. One hundred sixty six (28.9%) of study participants were attended primary school. Two hundred sixty two (45.6%) and 246 (42.7%) were followers of protestant and Orthodox religions, respectively. Two hundred forty seven (69%) of the mothers are currently living with their husband. One hundred seventy-three (30%) of the study participants were government workers. Two hundred thirty two (40.4%) of the respondents earn monthly income of greater than 1500 Ethiopian Birr (27 Ethiopian Birr = 1 USD). Three hundred eighteen (55.2%) of the respondents had family size of 3–6 (Table 1).

**Table 1:** Socio-demographic characteristics of the respondents of knowledge and attitude towards legalization of abortion, in Arba Minch town 2017 (*n* = 576)

Characteristic		Frequency(*n*)	Percent (%)
Age in year	15–19	9	1.6
	20–24	21	3.6
	25–29	86	15
	30–34	112	19.4
	35–39	167	29
	40–44	94	16.3
	45–49	87	15.1
Religion	Protestant	262	45.6
	Orthodox	246	42.7
	Muslim	53	9.1
	Others	15	2.6
Marital status	Married	247	69
	Single	77	13.3
	Divorced	40	7
	Widowed	62	10.7
Ethnicity**	Gamo	256	44.5
	Goffa	59	10.2
	Wolaita	87	15
	Konso	161	28
	Others	13	2.3
Educational status	Not educated	62	10.7
	Able to write & read	132	22.9
	Primary school	166	29
	Secondary school	26	4.4
	Higher education	190	33
Occupation	Government workers	173	29.9
	Merchant	150	26
	House wife	151	26.3
	Student	58	10.2
	Daily laborer	36	6.3
	Private worker	8	1.3
Monthly income in Ethiopian Birr.	Less than 500	43	7.3
	500–1500	172	29.9
	Greater than 1500	232	40.4
	Not known	129	22.4
Family size	Less than 3	183	31.8
	3–6	318	55.2
	Greater than 6	75	13

**Gurage, Zeyis, and Burji the respective frequencies were 4 for Gurage, 7 for Zeyis and 2 for Burji

### Abortion history

Among women included in the study 476(82.6%) had ever pregnant while 159(27.6%) had the history of unwanted pregnancy. One hundred twenty five (21.6%) of respondents have had induced abortion. From the total study participants about ninety two (73.5%) use private health institution as the place of abortion. Two hundred seventy one (47.1%) of women want to continue if they had unwanted pregnancy; while 158 (27.6%) women desire to terminate. Among the respondents, 372 (64.6%) were using family planning (Table 2).

**Table 2:** Abortion history of respondents of knowledge and attitude towards legalization of abortion, in Arba Minch town 2017 (*n* = 576)

Characteristics		Number	Percent
Ever been pregnant	Yes	476	82.6
	No	100	17.4
Ever had the unwanted pregnancy	Yes	159	27.6
	No	417	72.4
Ever had induced abortion	Yes	125	21.6
	No	451	78.4
Place of abortion	Patients home	6	4.8
	Hospital	3	2.4
	Abortionists home	17	13.2
	Health center	7	6
	Private health institution	92	73.5
If you have unwanted pregnancy any time what you do?	Continue and give birth	271	47.1
	Terminate	159	27.6
	I’m not sure	146	25.3
Have you ever used the contraceptive method	Yes	426	74
	No	150	26
Are you currently using family planning	Yes	372	64.6
	No	204	35.4

### The attitude of women toward legalization of abortion

Among women included in the study 323(56%) prefer abortion on demand to be legalized while 241 (41.9%) do not prefer to be legalized. Out of the respondents 327 (56.8%) were think that if abortion is legally allowed people can use the service. Three hundred forty (59%) of respondents do not use the service by themselves if abortion is legally allowed and 308(53.4%) also do not think that woman have the right to terminate their pregnancy. Two hundred seventy (46.8%) do not agree if women decided for some reason to terminate their pregnancy (Table 3).

**Table 3:** Attitude of respondents of attitude towards legalization of abortion, in Arba Minch town 2017 (*n* = 576)

Characteristics		Number	Percent
Do you prefer abortion on demand to be legalized?	Yes	323	56
	No	241	41.9
	I’m not sure	12	2.1
If abortion legally allowed, do you think people would use the service?	Yes	327	56.8
	No	249	43.2
If abortion legally allowed, do you use the service?	Yes	236	41
	No	340	59
Do you think women have the right to terminate the pregnancy if she wants?	Yes	247	43
	No	308	53.4
	I don’t know	21	3.6
If women decide for some reason to terminate their pregnancy do you agree or disagree	Agree	243	42.2
	Disagree	279	48.4
	I don’t mind	54	9.3

### Knowledge of respondents toward legalization of abortion

Among the women included in the study 187 (32.5%) had ever heard about safe abortion while 389(67.5%) had none. Out of respondents who had ever heard about save abortion 107(19%) were heard from their friends. Three hundred ninety six (69%) of respondents didn’t know about the complication of abortion while only 180(31%) knew. From the respondents only 135(23.4%) of women knew whether abortion was legal in Ethiopia but, majorities (67%) of respondents did not knew. From those respondents who knew about legalization of abortion in Ethiopia, 108(80%), 80(59%), 114(84.4%) and 18(13%) mentioned that abortion is legal if it is by incest, has a problem on mother; by rape and mother didn’t want respectively. One hundred seventeen (86.7%) of respondents believe that abortion was decided by women themselves while 18(13.3%) of them by doctor /health professionals. According to 93(69%) of respondents the time of abortion was before 3 months of pregnancy (Table 4).

**Table 4:** Knowledge of respondents of attitude towards legalization of abortion, in Arbaminch town 2017 (*n* = 576)

Variables		Frequency (*n*)	Percent (%)
Have you ever heard about safe abortion	Yes	187	32.5
	No	389	67.5
If yes from where you get the information	Mass media	52	9
	Health professional	17	3
	Relatives/family	12	2
	Friend	107	19
Do you know the complication of abortion?	Yes	180	31
	No	396	69
If yes, when (more than one answer is possible)*	If it is by incest	108	80
	If it is problem on mother	79	59
	If it is by rape	114	84.4
	If the mother doesn’t want	18	13
	If woman is less than 18	0	0
	women with mental problem	0	0
	women with Physical disable	0	0
Do you know abortion in demand is legal in Ethiopia?	Yes	135	23.4
	No	385	67
	I don’t know	56	9.6
Do you know who decide on abortion	Doctor /health professionals	18	13.3
	Women	117	86.7
	Another person	0	0
When is the preferable time to perform safe abortion	Before 3 months of pregnancy	93	69
	At any time during pregnancy	42	31

*Others person: anyone other than health professional and woman herself; might be her partner or family

### Factors associated with attitude toward legalization of abortion

All predictors of attitude toward legalization of abortion were entered into a logistic regression model and the final associated factors were identified. From those entered into the model, marital status, educational, pregnancy termination history and knowledge were statistically significant that affect the attitude of women toward legalization of abortion. The study revealed that single women and divorced were 81.9 and 93.1% times less likely had a good attitude as compared to married (Adjusted Odds Ratio (AOR) = .181, 95% Confidence Interval(CI): 0.377–0. 087) and (Adjusted Odds Ratio (AOR) =0.069, 95% Confidence Interval(CI): 0.062–0.460) respectively.

The attitude toward legalization of abortion among women who attend primary school was 3.666 times (AOR = 3.666, 95% CI: 1.772–7.581) and 3.431 times (AOR = 3.431, 95% CI: 1.083–10.87) more likely compare to those who attended higher education. Again, those who were illiterate and read & write were 4.804 and 11.258 times more likely good attitude than higher education (AOR = 4.804 and 11.26, 95%CI:1.453, 15.881 and 4.49, 28.227) respectively. Knowledge is a factor for attitude toward legalization of abortion. Those who answer, currently abortion on demand is illegal in Ethiopia 77.6% times (AOR = 0.224, 95% CI: .123–.409) less likely had a good attitude than those who answered I don’t know. But those who know abortion on demand is legal in Ethiopia were 1.84 times (AOR = 1.84, 95% CI: 1.137–2.976) more likely good attitude than those who don’t know (Table 5).

**Table 5:** Factors associated with the attitude of respondents of attitude towards legalization of abortion, in Arba Minch town 2017

		Attitude toward legalization of abortion		B	Sig.	AOR	95% C.I. for AOR	
		Positive	Negative				Lower	Upper
Marital status	Married	93	172		.000			
	Single	26	25	−1.707	.000	.181	.087	.377
	Divorced	22	5	−1.778	.000	.169	.062	.460
	Widowed	16	25	−.051	.879	.950	.489	1.844
Educational level	College & above	64	63		.000			
	Illiterate	4	37	1.569	.010	4.804	1.453	15.881
	Read and write	26	62	2.421	.000	11.26	4.490	28.227
	Primary school	53	58	1.299	.000	3.666	1.772	7.581
	Secondary school	10	7	1.233	.036	3.431	1.083	10.869
Pregnancy terminated	Yes	54	45	−1.717	.000	.180	.095	.339
	No	103	182	.033	.773	1.034		
Do you know, currently abortion on demand is legal or illegal in Ethiopia								
Don’t know		62	107		.000			
Illegal		38	87	−1.494	.000	.224	.123	.409
Legal		57	33	.609	.013	1.839	1.137	2.976

### Factors associated with knowledge toward legalization of abortion

All predictors of knowledge toward legalization of abortion were entered into a logistic regression model and the final associated factors were identified. From those entered into the model, marital status, ethnicity and family size were statistically significant for knowledge. The knowledge of women who were Konso, Wolaita and those who were other in ethnicity was 93, 86 and 95.3% less likely more knowledgeable about the legalization of abortion compared to women who were Gamo in ethnicity respectively. The study revealed that single women were about 95.5% times (AOR = .045, 95% CI: .013–0. 158) less likely good knowledge as compared to married women and also the knowledge among divorced were 99.2% times (AOR = 0.008, 95% CI: 0.002–0.040) less likely compared to who married. Similarly, women who have less than 3 and more than 6 children were about 71.5 and 59.6% times (AOR = 0.285, 95% CI: 0.145–0.561) and (AOR = 0.404, 95% CI: 0.174–0.939) less likely had knowledge than those who have 3–6 children respectively (Table 6).

**Table 6:** Factors associated with knowledge of respondents of attitude towards legalization of abortion, in Arbaminch town, 2017

Variables		Knowledge		Sig.	AOR	95% CI for AOR	
		Legal	Illegal			Lower	Upper
Constant			2.045	.000	7.732		
Ethnicity	Gamo	31	140	.000			
	Wolaita	17	41	.001	.140	.045	.438
	Konso	37	70	.000	.071	.029	.174
	Others	5	43	.001	.047	.007	.302
Marital status	Married	50	215	.000			
	Single	15	36	.000	.045	.013	.158
	Divorced	19	8	.000	.008	.002	.040
	Widowed	6	35	.428	.568	.141	2.296
Number of children	3–6 children	55	157	.009			
	Less than 3	29	93	.000	.285	.145	.561
	More than 6	6	44	.035	.404	.174	.939

## Discussion

The finding of our study showed that knowledge of women toward legalization of abortion was 23.4% which is low. The result was lower than study done in other part of the country. The study from Harari town revealed that about 35.7% of female students have knowledge towards the legislation of abortion. Again, the finding was much lower than the study done in Debra Markos hospital which was 92% [1]. Also, lower than the study conducted in other countries. The result was lower than study result in South Africa and Armenia which was 32% in South Africa [19] and 31% of women knew that, abortion is legal under any condition in Armenia [13]. The possible difference might be the difference in socio economic condition. But, the finding of this study was higher than study done in Zambia and Nepal. In Zambia the result was 16% [11]. In Nepal, from 1100 rural married women, only 15% knew about abortion law [13]. These findings clearly showed that the majority of women did not get information on their own affairs. Lack of knowledge is the result of lack of information. The causes of lower knowledge in the study area might be due to poor information dissemination to the target community. The result of systematic review showed that women who have knowledge of the legal status of abortion were less than 50% [20]. But, a study done in Latvia showed that more than half (53%) of women knew about the legalization of abortion [10, 19, 21]. In contradiction, this result was much higher than study done in Mizan Aman town of Ethiopia which was only 5.7% knew about the legalization of abortion [22]. This might be due to information dissemination problem throughout the country.

From those women who have good knowledge on the legalization of abortion majorities (84%) and (80%) of them believe it is legal if pregnancy was from rape/incest and from relative respectively. More than half (59%) of women, believes abortion is legal if it has problem on mothers as well as only 13% believe it is legally allowed for the mother if she don’t want.

Concerning the attitude of women; more than half of the respondents had a good attitude toward the abortion legalization while 42% do not. The result was consistent with the study done in the Mizan Aman town in which the attitude of women toward the legalization of abortion was 54.4% [22]. But, the result was somewhat higher than study done in Armenia and Debra Markos hospital which was 30 and 23% respectively [1, 13]. This difference might be due to the reality of the problem in the community. In Ethiopia, act of abortion has condemned almost by all religion and cultures. But, condemnation alone might not bring solution. More than half (57%) of the participants believe if service become legal, women can use the service but, 59% of women don’t think they will use by themselves even if abortion would be legal in Ethiopia. Almost half (53.3%) of respondents don’t think that women would have the right to terminate their pregnancy if the pregnancy fulfills the criteria. Again about 47% do not agree if women decided to terminate their pregnancy in any case. Therefore, the result showed that the majority (56%) of women had a positive attitude toward the legalization of abortion; but still large proportion of women have negative attitude toward the legalization of abortion. This perception of the community shows still need an intervention. In Ethiopia, since 2004 abortion has been legalized under some circumstances. But only less than 6% used public health facilities and about 73% uses private clinics in this finding; the possible reason might be the low knowledge and problem related to the attitude. Changing community knowledge and attitudes might be challenging; particularly when the topic is stigmatized. Additional intervention be needed to improve access to safe abortion service and other reproductive services for women at the community level.

Nearly 40 years after India legalized abortion, Indian women continue to be unaware that safe abortion service was given at public health facilities or was unable to access it. Although abortion has been legal in India for decades, unsafe abortions were estimated to be 90% [18]. The underlying reason might be the attitude related to the issue. In our case, East Africa, in particular, has one of the world’s highest rates of maternal mortality linked to complications from unsafe abortions. Over 50% of all women seeking abortions in Ethiopia do so outside the reach of trained medical professionals and outside of health facilities even after the legalization of safe abortion service [6]. The reason might be due to stigma and the wrong belief of the community toward abortion which enforces women to choose secrecy over safety.

In our study, ethnicity, marital status and family size were the socio demographic factors significantly associated with knowledge. For attitude, marital statuses, level of education as well as knowledge were associated factors. The same with the study done in Debra Markos hospital and Mizan Aman town where the knowledge was the associated factors [1, 22].This result was in line with the study done in Harari and Zambia; where age, religion and marital status were a factor, but in our study age and religion were not significant [11, 21]. But, accessibility to abortion service was a factor for legalization of abortion in Zambia but not in our case [11]. Again study done in Mizan Aman town, the preference of termination was a factor for the knowledge of abortion; but here in our study it was not an associated factor [22].

The study showed that educational status, marital status and having knowledge about the legalization of abortion has a statistically significant association with an attitude. The result was in line with the study conducted in Mizan Aman town and Yirgalem south nation nationality of Ethiopia and other parts of Africa [11, 12, 19].

## Conclusion

In conclusion, our study indicated that knowledge of women about the legalization of abortion was low and more than half of women had positive attitude to the legalization of abortion. But, still immense proportion of women (42%) have negative attitude toward the legalization of abortion. Moreover, Ethnicity, marital status, and the number of children were strong predictors of knowledge while education, history of pregnancy termination and knowledge were the predictor of attitude toward legalization of abortion. Thus, it was recommended that the concerned body should give attention to awareness creation and give comprehensive health education and information should be given on a local basis.

## References

[1] Adera A, Kassaw MW, Yimam Y, Abera H, Dessie G (2016). Assessment of knowledge , attitude and practice women of reproductive age group towards abortion Care at Debre Markos Referral Hospital, Ethiopia. Sci J Public Health.

[2] Ipas (2010). Facts on Unintended Pregnancy and Abortion in Ethiopia.

[3] Prata N, Holston M, Fraser A, Melkamu Y (2013). Contraceptive use among women seeking repeat abortion in Addis Ababa, Ethiopia. Afr J Reprod Health.

[4] Mekuriaw S, Mesay R (2015). Knowledge , Attitude and Practice towards Safe Abortion among Femalestudents of Mizan-Tepi University, South West Ethiopia. Womens Health Care.

[5] Vekemans M (2005). First trimester abortion guidelines and protocols; Parenthood Federation, International planned.

[6] Mesce D, Clifton D. Abortion facts and figures 2011. PRB’s website 2011;5–64.

[7] Otsea Karen, Benson Janie, Alemayehu Tibebu, Pearson Erin, Healy Joan (2011). Testing the Safe Abortion Care model in Ethiopia to monitor service availability, use, and quality. International Journal of Gynecology & Obstetrics.

[8] Central Ststistics Agency. Ethiopia demographic and health survey. CSA 2016 p. 46–59.

[9] Wada T (2008). Abortion law in ethiopia : a comparative perspective. Mizan Law Rev.

[10] Melgalve I, Lazdane G, Trapenciere I, Shannoo C, Bracken HWB (2005). Knowledge and attitudes about abortion legislation and abortion methods among abortion clients in Latvia. Eur J Contracept Reprod Health Care.

[11] Cresswell JA, Schroeder R, Dennis M, Onikepe O, Bellington V, Maurice M (2016). Women ’ s knowledge and attitudes surrounding abortion in Zambia : a cross-sectional survey across three provinces. BMJ Open.

[12] Bitew S, Ketema S, Worku M, Hamu M, Loha E (2013). Knowledge and attitude of women of childbearing age towards the legalization of abortion , Ethiopia. J Sci Innov Res.

[13] Chong E, Tsereteli T, Vardanyan S, Avagyan G, Winikoff B (2009). Knowledge atttude and practice of abortion amongwomen and doctors in Armenia. Eur J Contracept Reprod Health Care..

[14] World Health Organization(WHO). Safe abortion : http://www.who.int; second edited. 2012; 6–134.

[15] Benson J, et al. Meetingwomen’s needs for postabortion family planning: framing the questions. Issues in Abortion Care. Int. J. Gynecol. Obstet. 1992;2(2). https://www.popline.org/node/325274.

[16] Muzeyen R., Ayichiluhm M., Manyazewal T. (2017). Legal rights to safe abortion: knowledge and attitude of women in North-West Ethiopia toward the current Ethiopian abortion law. Public Health.

[17] Family health department Federal Democratic of Ethiopia. Technical and Procedural Guidelines for Safe Abortion Services in Ethiopia. web. 2006. p. 12–26.

[18] Namrata S, Sumitra Y (2015). The study of knowledge , attitude and practice of medical abortion in women at a tertiary Centre. IOSR J Dent Med Sci.

[19] Morroni C, Myer L, Tibazarwa K (2006). Knowledge of the abortion legislation among south African women_ a cross-sectional study. BMC Reprod Health.

[20] Assifi AR, Berger B, Tunçalp Ö, Khosla R, Ganatra B (2016). Women ’ s awareness and knowledge of abortion Laws : a systematic review. PLoS One.

[21] Geleto A, Markos J (2015). Awareness of female students attending higher educational institutions toward legalization of safe abortion and associated factors , Harari region , eastern Ethiopia : a cross sectional study. Reprod Health.

[22] Mara AM, Ayenew M, Haftu H, Aregay B (2017). Assessment of knowledge and attitudes of men and women aged between 15-49 years towards legalization of induced abortion in Mizan Aman town. J Women's Health Care.

